# Induction of Epigenetic Alteration by CPUK02, An Ent- kaurenoid Derivative of Stevioside

**Published:** 2017

**Authors:** Pooneh Mokarram, Zeinab Mohammadi, Saeid Khazayel, Zhang Dayong

**Affiliations:** 1. Gasteroenterohepatology Research Center, Nemazee Hospital, Faculty of Medicine, Shiraz University of Medical Sciences, Shiraz, Iran; 2. Department of Biochemistry, Faculty of Medicine, Shiraz University of Medical Sciences, Shiraz, Iran; 3. Drug Research Institute, China Pharmaceutical University, Jiangsu, China

**Keywords:** 5-AZA, Colorectal neoplasm, DNMT, Epigenetic, Methylation

## Abstract

**Background:**

Dietary polyphenols, such as those found in green tea and red wine, are linked to antitumor activity. They are known to influence many signaling pathways epigenetically within the human body. In this regard, CPUK02 (15-Oxosteviol benzyl ester) is a new ent-kaurenoid derivative of stevioside and exhibits strong anti-cancer activity *in vitro* and *in vivo*. Nowadays, the role of epigenetics in cancer has been the subject of intensive study and DNA methylation targeting represents a relevant strategy for cancer treatment. There are no reports regarding the effects of CPUK02 on epigenetic alterations in colorectal cancer cell line. This study was an attempt to compare CPUK02 with 5-AZA as DNMT inhibitor agent and evaluate whether it can induce its anti-cancer effects via altering the level of DNMT3b mRNA, MGMT and SFRP2 methylation pattern in HCT 116 cell line.

**Methods:**

To evaluate DNMT3b expression, DNMT3B mRNA levels in HCT116 CRC cell line were quantified by real-time reverse-transcriptase Polymerase Chain Reaction (PCR) assay after 24 *hr* of incubation time with CPUK02 and 5-AZA. In addition, the methylation patterns of 2 CpG islands in this cell line were examined by methylation-specific PCR methods.

**Results:**

CPUK02 surprisingly, decreased the DNMT3b mRNA level. The average expression levels of DNMT3b in HCT116 treated with CPUK02 and 5-AZA relative to the GAPDH expression level in control were 0.16 and 0.5%, respectively. Furthermore, CPUK02 could decrease the methylated allele of MGMT and SFRP2 genes in HCT 116 after 24 *hr*.

**Conclusion:**

In this study, positive correlation was found between mRNA expression of DNMT3b and gene promoter hypermethylation after treatment with CPUK02 and 5-AZA. Our data confirmed that CPUK02 like 5-AZA exhibits demethylating properties.

## Introduction

Colorectal Cancer (CRC) is a multistep process with accumulation of genetic and epigenetic errors which causes a normal cell transformation to an invasive tumor cell 1.

Three distinct pathways including chromosomal instability, microsatellite instability, and CpG island gene methylation pathways have been recognized in colon cancer initiation and progression. Alterations in DNA methylation patterns are the best understood epigenetic cause of the disease which occurs usually *via* either hypomethylation of global DNA or hypermethylation of tumor suppressor genes 2. DNA methylation is performed by at least two DNA methyltransferases (DN MT; 3a and 3b) and maintained by DNMT1 3. Overexpression of DNMTs has been also detected in a variety of malignancies, including lung, prostate, and colorectal tumors 4.

DNA hypermethylation is also associated with gene silencing and is often observed in CpG islands of cancer-related genes. Transcriptional silencing by the hypermethylation of CpG islands is an early event in tumor progression. In this regard, researchers suggest that hypermethylation of O6-methylguanine-DNA methyltransferase (O6-MGMT) is a pacemaker for certain gene mutations such as K-ras 5. On the other hand, aberrant methylation in genes related to WNT signaling pathway has crucial roles for cancer progression especially in CRC 6. Among specific genes in WNT signaling, SFRP2 methylation is considered as a marker with high sensitivity and specificity in serum and stool for colorectal adenomas and CRC screening 7–9. 5-Aza-CR and 5-Aza-CdR potentially inhibit the DNA methyltransferases (DNMTs) 10–13. Nowadays, therapeutic targeting *via* the DNA methylation machinery has been the subject of intensive study 14.

Recently, natural compounds, such as curcumin, Epigallocatechin Gallate (EGCG) and resveratrol, have been considered to increase sensitivity of cancer cells to conventional agents and induce tumor growth inhibition *via* epigenetic mechanisms 15. One of the plants that has recently attracted the attention of researchers is stevia rebaudiana bertoni and its glycoside compounds. Among stevia derivatives, isosteviol and derivative compounds inhibit human cancer cell growth 16. Furthermore, only isosteviol potently suppresses both mammalian DNA polymerases and human DNA topoisomerase 17,18. Paul *et al* also reported that stevioside is a potent inducer of apoptosis *via* intracellular ROS generation in MCF7 breast cancer cell line 19. In this regard, the Chinese researcher made a new ent-kaurenoid derivative of stevioside known as CPUK02 20. A large number of ent-kaurane diterpenoids isolated from different natural plants have been reported to display anticancer activities 21. The anticancer activity of CPUK02 was evaluated in *in vitro* and *in vivo* models. CPUK02 strongly inhibits cell proliferation and induces apoptosis in several human cancer cell lines. Furthermore, CPUK02 demonstrates its anti-tumor effects much better than fluorouracil (5-FU) in mouse xeno-graft cancer model 22.

Lack of information about the effects of CPUK02 on epigenetic alteration in colorectal cancer mandates more research in this field. Therefore, the aim of this study was to further elucidate the anti-cancer effects of CPUK02 *via* epigenetic alteration. In this regard, the level of DNMT3B mRNA expression, MGMT and SFRP2 methylation pattern as representative of epigenetic changes in HCT 116 cell line were assessed.

## Materials and Methods

The human colon cancer cell line, HCT 116 was obtained from the National cell bank of Iran (Pasteur Institute, IRAN). Cell culture medium, penicillin, streptomycin medium supplement, glutamine and fetal bovine serum were obtained from Gibco Life Technologies (UK). Tripure Isolation Reagent was purchased from Roche Applied Sciences (USA).

DNA Synthesis Kit was purchased from Fermentas, EU. SYBR green DNA PCR Master Mix was purchased from the Applied Biosystem (ABI) Company, (Foster City, CA USA). CPUK02 was kindly provided as a gift from Drug Research Institute, China Pharmaceutical University.

### Cell line

The human colon cell line was cultured in RPMI 1640 medium supplemented with 10% fetal bovine serum and incubated at 37°*C* in a humidified 5% (*v/v*) CO_2_ incubator.

### Treatment with CPUK02

Cells were seeded for 12 *hr* and then washed with PBS and medium was replaced with serum-free RPMI medium. Finally, non-growing, confluent cells were incubated with CPUK02 and 5-aza dissolved in DMSO to a final concentration of 4.5 *μM* and 1 *μM*, respectively for 24 *hr*. Optimum concentrations were determined according to MTT assay. At the end of the incubation, cells were harvested for RNA and DNA isolation.

### Methylation specific-PCR analysis

DNA was extracted from cells according to the standard phenol/chloroform method 23. The status of promoter methylation of the MGMT-B, and SFRP2 genes was determined by Methylation –Specific PCR (MSP-PCR). The sequences of primers and annealing temperatures used for amplification of the promoter regions of genes are listed in table 1.

**Table 1.** Primer sequence of SFRP2 and MGMT genes

**Gene**		**Primer sequence**	**Annealing temperature, °C**	**Product size (*bp*)**
**MGMT**				
	Primer sequence (5–3)	MF: GGTCGTTTGTACGTTCGC	59	M: 127;U:127
		MR: TAACCCTTCGACCGATACAA		
		UF: GTAGGTTGTTTGTATGTTTGT		
		UR: TAACCCTTCAACCAAAAACC		
**SFRP2**				
	Primer sequence (5–3)	MF: TGCGTGTTTTTTATTTTCGTAGTTCGC	59	M:138;U145
		MR: CCCTAAATACCGCCGCTCGCCCG		
		UF: GTTTTGTGTGTTTTTTATTTTTGTAGTTTGT		
		UR: TCCCCTAAATACCACCACTCACCCA		

The genes promoter methylation status was determined by chemical treatment of DNA samples with sodium bisulfite and subsequent MS-PCR as previously described 24.

In every reaction, DNA from peripheral blood lymphocytes was considered as a negative control. PCR products were analyzed by electrophoresis on 2% agarose gel.

### RNA extraction and semi-quantitative PCR

The total RNA was extracted from the treated cells using the TRIZOL Reagent Kit, according to the manufacturer’s instructions. The RNA concentration was quantified by measuring the absorbance at 260 *nm* in a spectrophotometer. Ratios of absorption (260/280 *nm*) of all samples were between 1.8 and 2.0. RNA samples were subjected to electrophoresis through a 1.4% agarose-formaldehyde gel to verify their integrity. The total RNA samples were stored at −80°*C* until analysis. The expression of DNMT3b mRNA was determined by semi-quantitative Reverse Transcription Polymerase Chain Reaction (RT-PCR). Two *μg* of total RNA were reverse transcribed by incubation at 37°*C* for 1 *hr* in a 25 *μl* reaction mixture consisting of 100 *U M* reverse transcriptase, 8 *U* RNase inhibitor, 0.5 *μg* of oligo (dT), 50 *mmol/L* Tris-HCl (pH=8.3), 3 *mmol/L* MgCl_2_, 75 *mmol/L* KCl, 10 *mmol/L* DDT, and 0.8 *mmol/L* each dNTP. The reaction was terminated by heating at 95°*C* for 5 *min* and quickly cooling on ice. Two *μl* of the RT reaction mixture were used for PCR in a final volume of 25 *μl*, containing 2.5 *μl* of 10×PCR buffer, 2 *μl* of each 2.5 *mmol/L* dNTPs mixture, 1 unit of Taq DNA polymerase, and 10 *pmol/L* of each forward and reverse primer. The conditions of PCR amplification were as follows: one cycle at 95°*C* for 5 *min*, 33 cycles at 95°*C* for 30 *s*, 60°*C* for 40 *s* and 72°*C* for 1 *min*, and a final extension cycle at 72°*C* for 10 *min*. The PCR-amplified fragments were run alongside molecular weight markers on 2% agarose gels stained with gel red. The experiments were repeated twice. The primers and PCR conditions for DNMT3b, GAPDH are listed in table 2. 232 and 113 *bp* amplicons were expected upon performing PCR for GAPDH and DNMT3b, respectively.

**Table 2.** Primer sequence of DNMT3b and GAPDH genes

**Gene**	**Primer sequence**	**Annealing temperature, °*C***	**Product size (*bp*)**
**DNMT 3B**	F: GGCAAGTTCTCCGAGGTCTCT	60	113
	R: TGGTACATGGCTTTTCGATAGGA		
**GAPDH**	F: CGACCACTTTGTCAAGCTCA	60	228
	R: AGGGGTCTACATGGCAACTG		

### Quantitative real-time PCR

Real-time PCR was carried out using the ABI real time PCR 7500 system. The PCR reaction mixture contained 2 *μl* of cDNA (tenfold diluted), 0.5 *μl* of 5 *mmol/L* solutions of each of the forward and reverse primers and 10 *μl* of SYBR green DNA PCR Master Mix in a total volume of 20 *μl*. All incubations included an initial denaturation step at 95°*C* for 10 *min* and 30 cycles (15 *sec* at 95°*C* and 30 *sec* at 60°*C*) subsequently. A melting curve analysis was achieved by performing 70 cycles of 10 *s* with a temperature increment of 0.5°*C/cycle* starting from 60°*C*. Efficiency of amplification was measured by the slope of a standard curve. Data were analyzed by using the 7500 Software v2.0.1. The relative expression level (fold changes) of DNMT3b gene was calculated by the 2^−ΔΔCT^ formula. GAPDH was also considered as the internal control. Expression levels of target gene were normalized using the GAPDH as the housekeeping control gene.

### Statistical analysis

Statistical analysis was performed using one-way analysis of variance (ANOVA) followed by post-hoc Dunnett multiple comparison tests using SPSS [Version 16; SPSS, Chicago, IL, USA]. The values of p< 0.05 were considered statistically significant.

## Results

### Methylation of SFRP2 and MGMT genes

**Figure 1.**
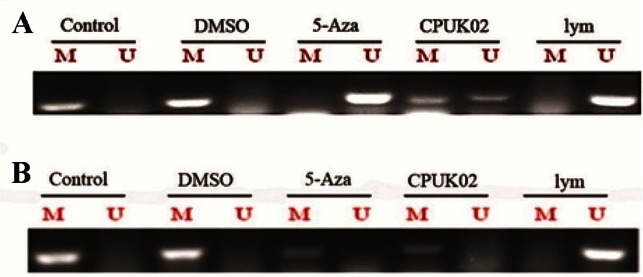
Representative example of MSP-PCR for promotor methylation analysis of genes *SFRP2* A) and MGMT; B) and in HCT 116 cells. The presence of a visible PCR product in those lanes marked U indicates the presence of unmethylated genes; the presence of a product in those lanes marked M indicates the presence of methylated genes in control, cells treated with DMSO as vehicle as well as 5-AZA and CPUK02. Unmethylated lymphocytes (lymphocytes) DNA was used as the negative control.

The methylated and unmethylated sense/antisense primers for MGMT produced a 127 *bp* fragments. However, the methylated and unmethylated sense/antisense primers for SFRP2 produced 138 and 145 *bp* fragments, respectively. In this study, it was observed that CPUK02 has a positive effect on SFRP2 and MGMT methylation pattern in HCT 116 cells. CPUK-02 and 5-aza had a similar effect on the methylation of MGMT gene promoter. As shown in figure 1B, both of them moderately decreased methylated allele. However, according to our results (Figure 1A), 5-aza completely inhibited SFRP2 methylation but CPUK02 moderately did.

### DNMT3b expression analysis

**Figure 2.**
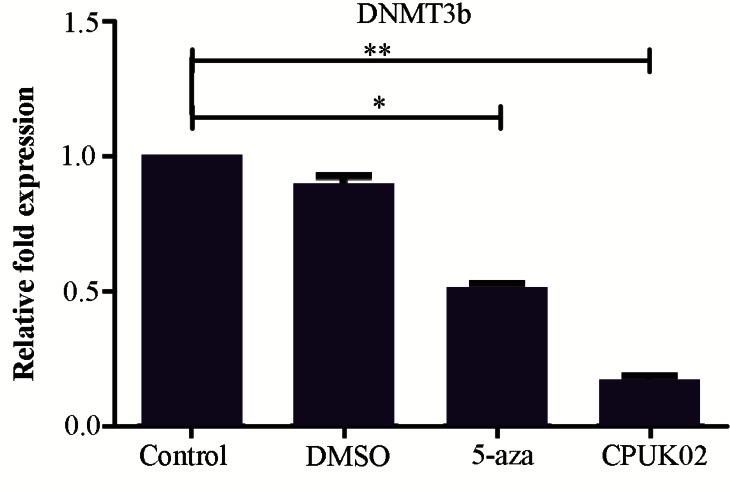
The effect of CPUK02 on DNMT3b expression. CPUK02 and 5-aza were able to decrease mRNA level of *DNMT3b* gene in comparison with control group in HCT 116 cells.

The presence of appropriate bands for DNMT3b (113 *bp*) and GAPDH (228 *bp*) amplicons was confirmed by semi RT-PCR. Real time PCR results in figure 2 showed that CPUK02 and 5-aza were able to decrease mRNA level of DNMT3b gene compared with control group in HCT 116 cells. The average expression levels of DNMT3b in HCT116 treated with CPUK02 and 5-AZA relative to the GAPDH expression level in control were 0.16 and 0.5%, respectively.

## Discussion

For the first time, the epigenetic role of CPUK02 in HCT 116 cells was shown. CPUK02 as an ent-kaurenoid compound exhibited strong effect upon DNMT3b mRNA level and methylation status of two important genes; MGMT and SFRP2 in CRC.

Researchers showed that CPUK02 can induce the apoptosis in different cancer cell lines via p53 22. However, there is no data to address the effects of CPUK02 on epigenetic alteration in colorectal cancer. Pierre-Olivier and colleague have found that DNMTs are integral part of the p53 protein network. On the other hand, the levels of DNMTs, especially DNMT3a and DNMT3b, are often increased in various cancer tissues and cell lines such as HCT 116 cells 25. Therefore, this phenomenon may increase hypermethylation of tumor suppressor genes in a variety of malignancies. Although cytosine promoter methylation is accomplished by five mammalian DNMTs (DNMT1, DNMT2, DNMT3a, DNMT3b, and DNMT3L), but in contrast to DNMT1, DNMT3a and DNMT3b are able to do de novo DNA methylation during normal development. DNMT1 is also required for maintenance of methylation patterns 26.

Regarding the importance of DNMT3b, several studies demonstrated that DNMT3b is overexpressed at higher frequency than DNMT3a and DNMT1 in CRC and breast cancer 27–29. In addition, DNMT3B overexpression is associated with CIMP-high phenotype in colorectal cancer 30. Furthermore, direct interaction between Dnmt3a and/or Dnmt3b and transcription factors provides a molecular mechanism which leads to DNA hyper methylation 31.

In this regard, Zhang *et al* showed that DNMT1, DNMT3b protein level decreased in HCT116 cells treated with DNA-damaging drug doxorubicin as the apoptosis-inducible agent 32. Furthermore, Qiang Li *et al* showed that 5-aza-2-deoxycytidine inhibited both DNMT1 and DNMT3B protein expression in breast, colon, and other types of cancer cells 33. Our data showed that CPUK02 was also able to decrease mRNA level of DNMT3b gene in HCT 116 cells. So, CPUK02 mimics the role of 5-AZA to decrease mRNA level of DNMT3b. However, considering CPUK02 as an epigenetic drug, further investigation is needed.

In the next part of our study, the effect of CPUK02 on the methylation status of two key genes in colorectal cancer initiation and progression was evaluated.

SFRP2 as a negative regulator of Wnt signaling has important implications in tumorigenesis, and its down regulation has been correlated with CRC 34–36. It has been also postulated that lack of MGMT expression increases the spontaneous G:C.A:T mutation rate in tumors *in vivo*. In addition, MGMT methylation is also a central member in cancer development from normal adenoma to carcinoma 37.

According to the importance of SFRP2 and MGMT role in cancer progression, promoter methylation of these two genes was investigated in HCT 116 treated with CPUK02. It was revealed that CPUK02 and 5-AZA had a similar effect on methylation pattern of MGMT gene. Both of them induced moderate demethylation of MGMT gene. In case of SFRP2, it was shown that 5-aza completely restored unmethylated allele but CPUK02 moderately did.

In this case, the ER-negative breast cells were also treated with demethylating agent 5-aza and CPUK02. As shown in supplement figure, our results showed that CPUK02 like 5-AZA may effectively restore ER aberrant hypermethylation. Thus, inactivation of ER gene has a close relationship with the abnormal methylation of ER gene promoter which can be restored by 5-AZA 38 and probably CPUK02.

**Supplement figure.**
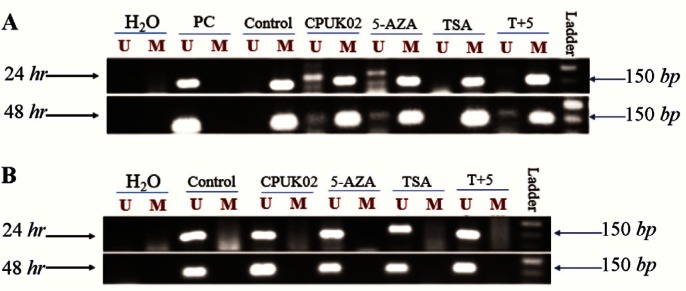
Representative example of MSP-PCR for pro-motor methylation analysis of ER1 in MDA_MB231 breast cancer cell line. Positive control: MCF_7 cell line, control: MDA_MB231 cell line. Both cell lines were treated under two conditions (24 *hr* and 48 *hr*).

However, 5-aza and CPUK02 were not able to completely restore the unmethylated pattern of MGMT gene in CRC cell line. In this regard, Wodarz *et al* revealed that methylation rate of MGMT gene in HCT-116 cell line is slower than methylation rate of SFRP2 gene 39. Also, they showed that MGMT promoter methylation in HCT 116 cell line started with a delay. Given this finding, it can be concluded that remethylation rate of MGMT gene will be slower than SFRP2. Therefore, increasing the incubation time during treatment from 24 *hr* to 48 might help the unmethylated allele of MGMT to appear. This experiment was performed on HCT 116 cells for longer time and the same results were obtained which are seen in ER-negative breast cells.

Recent researchers showed that epigenetic pharmaceuticals could be a replacement or adjuvant therapy for currently accepted treatment methods such as radiation and chemotherapy, or could sensitize cells to the current treatments 40. In this regard, although 5-Aza is the most potent demethylating agent in clinical usage, general side effects led to efforts for finding new DNA methylation inhibitors with greater potency and low cytotoxicity.

Recently, natural compounds, such as curcumin, EGCG, and resveratrol, have been shown to increase sensitivity of cancer cells to conventional agents via alteration in epigenetic mechanisms, which may lead to tumor growth inhibition 41. It has been shown that the epigenetic control of the proto-onco regions and the tumor suppressor sequences by conformational changes in histones directly affects the formation and progression of cancer 42–44. CPUK02 as a new semi natural compound which exhibits strong anti-cancer effect in *in vitro* and *in vivo* models might also manipulate epigenetic pathways and our data confirmed that CPUK02 like 5-AZA exhibits demethylating properties.

## Conclusion

According to our data, CPUK02 is a promising molecule with epigenetic/anti-cancer properties and it might be a front-runner candidate for new pharmaceutical targets. However, little information exists about performance of CPUK02 and further investigations are needed to introduce it as a new epigenetic drug.
